# Long noncoding RNAs as therapeutic targets to overcome chemoresistance in ovarian cancer

**DOI:** 10.3389/fcell.2022.999174

**Published:** 2022-08-29

**Authors:** Linjiao Chen, Jie Wang, Qian Liu

**Affiliations:** Department of Reproductive Medicine, Department of Prenatal Genetics, First Hospital of Jilin University, Changchun, China

**Keywords:** lncRNA, epigenetic, chemoresistance, ovarian cancer, cisplatin, paclitaxel, target

## Abstract

Long noncoding RNAs (lncRNAs) have been characterized to play an essential role in ovarian tumorigenesis via controlling a variety of cellular processes, such as cell proliferation, invasion, apoptotic death, metastasis, cell cycle, migration, metabolism, immune evasion, and chemoresistance. The one obstacle for the therapeutic efficacy is due to the development of drug resistance in ovarian cancer patients. Therefore, in this review article, we describe the role of lncRNAs in chemoresistance in ovarian cancer. Moreover, we discuss the molecular mechanism of lncRNAs-involved drug resistance in ovarian cancer. We conclude that lncRNAs could be useful targets to overcome chemoresistance and improve therapeutic outcome in ovarian cancer patients.

## Introduction

Ovarian cancer is one of common malignancies in females (Giaquinto et al., 2022; Siegel et al., 2022). The key obstacle for the therapy outcome is the development of drug resistance in ovarian cancer patients (Ortiz et al., 2022). Genetic and epigenetic changes have been known to participate in chemotherapy resistance in a variety of cancers, including ovarian cancer (Meng et al., 2021; Zhang et al., 2022; Zhao et al., 2022). LncRNAs are remarkably involved in carcinogenesis via regulating a variety of cellular processes, such as cell proliferation, invasion, apoptotic death, metastasis, cell cycle, migration, metabolism, immune evasion (Jiang et al., 2021; Chen et al., 2022a; Chen et al., 2022b; Liu and Shang, 2022). LncRNAs can epigenetically regulate gene expression and take part in drug resistance (Jiang et al., 2020a; Xie et al., 2022). LncRNAs bind to proteins and nucleic acids and regulate the expression of tumor suppressive genes and oncogenes through working as decoys, scaffolds, or enhancers (Liu et al., 2021; Statello et al., 2021).

Noncoding RNAs have been involved in drug resistance and can become the therapeutic targets for cancer therapy (Lan et al., 2021; Xie et al., 2021; Liu et al., 2022). Drug resistance is developed due to decreased drug uptake, enhanced drug efflex, dysregulated drug metabolism, and drug compartmentalization. In addition, dysregulation of DNA damage repair and decreased sensitivity to apoptosis also lead to drug resistance. A ferroptosis and iron-metabolism-related lncRNA profile has been reported for prediction of therapeutic responses by analysis of integrated clinical data and omics data in ovarian cancer (Feng et al., 2022). LncRNAs have been emerged in regulating drug sensitivity in ovarian cancer (Tripathi et al., 2018; Abildgaard et al., 2019; Wambecke et al., 2020; Wambecke et al., 2021). In the following sections, we will describe the role of various lncRNAs in regulation of cisplatin, paclitaxel, 5-FU, rapamycin and carboplatin resistance.

## LncRNAs regulate cisplatin resistance

Cisplatin is platinum-based drug, which has been widely used for the therapy of ovarian cancer patients (Song et al., 2022). One study showed that lncRNA PVT1 was elevated in cisplatin resistant ovarian cancer tissues, while lncRNAs TUG1 and MEG3 were decreased in ovarian cancer patients with cisplatin resistance (El-Khazragy et al., 2020). Several lncRNAs have been reported to promote the cisplatin resistance in ovarian cancer, including ACTA2-AS1, CCAT1, CRNDE, HOTAIR, HOXA11-AS, TCF7, PANDAR, PART1, MALAT1, UCA1, WDFY3-AS2. However, numerous lncRNAs have been found to inhibit the cisplatin resistance in ovarian cancer, such as LINC01508 and GAS5.

## LncRNAs promote cisplatin resistance

### LncRNA ACTA2-AS1

The high expression of lncRNA ACTA2-AS1 was found in cisplatin-resistant A2780 and SKOV3 cells. Silencing of lncRNA ACTA2-AS1 in cisplatin-resistant ovarian cancer cells reduced cell proliferation (Lin et al., 2022). Moreover, lncRNA ACTA2-AS1 can sponge the expression of miR-378a-3p, which increased the expression of Wnt5a. Knockdown of miR-378a-3p reversed lncRNA ACTA2-AS1 knockdown-mediated inhibition of cisplatin-resistant cell viability (Lin et al., 2022). Consistently, miR-378a-3p alleviated cell resistance to cisplatin via suppression of Wnt5a. Hence, lncRNA ACTA2-AS1 boosted cisplatin resistance via sponging miR-378a-3p and targeting Wnt5a in ovarian cancer cells (Lin et al., 2022).

### LncRNA CCAT1

LncRNA CCAT1 has been known to involve in cisplatin resistance in ovarian cancer cells (Wang et al., 2020a). Cisplatin-resistant ovarian cancer cells had the higher expression of CCAT1. Silencing of CCAT1 triggered cisplatin-mediated apoptosis via regulation of Bax, survivin and Bcl-1. Notably, CCAT1 can bind to miR-454 and increase the expression of survivin (Wang et al., 2020a). Hence, CCAT1/miR-454/survivin pathway contributed to cisplatin resistance in ovarian cancer (Wang et al., 2020a).

### LncRNA CRNDE

Wu et al. reported that lncRNA colorectal neoplasia differentially expressed gene (CRNDE) promoted cisplatin resistance via regulation of SRSF1/TIA1 signaling pathway in ovarian cancer (Wu et al., 2022a). Cisplatin resistant ovarian cancer cells have a higher expression of lncRNA CRNDE compared with control cells. Overexpression of lncRNA CRNDE caused cisplatin resistance, whereas depletion of lncRNA CRNDE sensitized cancer cells to cisplatin *in vitro* and *in vivo* (Wu et al., 2022a). Moreover, lncRNA CRNDE increased SRSF1 expression and subsequently elevated TIA1 expression, which caused cisplatin resistance in ovarian cancer (Wu et al., 2022a).

### LncRNA HOTAIR

LncRNA HOTAIR had a lower expression in benign ovarian tissues compared with ovarian tumor samples (Wang et al., 2015). A higher expression of lncRNA HOTAIR was observed in late stage malignant tumors compared with the early stage samples in ovarian cancer (Wang et al., 2015). The higher expression of HOTAIR was also observed in cisplatin-resistant SKOV3 cells than cisplatin-sensitivity control. Downregulation of HOTAIR retarded cell proliferation and migratory ability and reversed cisplatin resistance in cisplatin-resistant SKOV3 cells (Wang et al., 2015). Similarly, silencing of lncRNA HOTAIR abolished cisplatin resistance via suppression of miR-138-5p-involved EZH2 and SIRT1 in ovarian cancer cells (Zhang et al., 2020a). Zhang et al. found that HOTAIR kept the stemness features by sponging miR-206 and increasing the expression of T-box transcription factor 3 (TBX3) in ovarian cancer stem cells, contributing to cisplatin resistance (Zhang et al., 2020b). Moreover, HOTAIR promoted cisplatin resistance via regulation of DNA damage response and senescence by activation of NF-κB signaling pathway via suppression of IκBα in ovarian cancer cells (Ozes et al., 2016). Consistently, depletion of HOTAIR reduced cisplatin resistance via attenuating cisplatin-mediated autophagy in ovarian cancer (Yu et al., 2018).

### LncRNA HOXA11-AS

Knockdown of lncRNA HOXA11-AS suppressed cell proliferation and overcame cisplatin resistance in ovarian cancer (Chen et al., 2022c). Cisplatin-resistance ovarian cancer cells had a higher expression of lncRNA HOXA11-AS compared with normal cells. Silencing of lncRNA HOXA11-AS in ovarian cancer cells suppressed cell viability, invasion and migration, but promoted apoptosis (Chen et al., 2022c). Knockout of lncRNA HOXA11-AS increased cisplatin sensitivity in cisplatin-resistant ovarian cancer cells. LncRNA HOXA11-AS depletion elevated cellular autophagy in ovarian cancer cells (Chen et al., 2022c).

### LncRNA TCF7

LncRNA TCF7 has been found to enhance cell viability, migration, invasion via modulation of ITGB8 in epithelial ovarian cancer (Su and Huang, 2021). Moreover, lncRNA TCF7 promoted the stemness of ovarian cancer cells via acceleration of spheres formation by promotion of CD44 and CD133 expression. Furthermore, lncRNA TCF7 accelerated cisplatin resistance in epithelial ovarian cancer cells (Su and Huang, 2021). Molecular experimental data showed that lncRNA TCF7 promoted the expression of ITGB8 and exerted its oncogenic function in ovarian cancer (Su and Huang, 2021).

### LncRNA PANDAR

LncRNA PANDAR participated in the drug resistance in ovarian cancer cells (Wang et al., 2018a). Cisplatin treatment induced higher expression levels of lncRNA PANDAR than paclitaxel and doxorubicin exposure in ovarian cancer. In cisplatin-sensitive ovarian cancer cells displayed lower expression of lncRNA PANDAR compared with cisplatin-resistant groups (Wang et al., 2018a). Moreover, overexpression of lncRNA PANDAR elevated the tumor growth and cell survival after cisplatin treatment, whereas downregulation of lncRNA PANDAR retarded tumor growth (Wang et al., 2018a). PANDAR interacted with SFRS2, a key factor to negatively govern p53 phosphorylation, leading to inhibition of PUMA in ovarian cancer (Wang et al., 2018a).

### LncRNA PART1

LncRNA PART1 participated in promotion of resistance of cancer cells to cisplatin exposure (Yang et al., 2021). LncRNA PART1 was highly expressed in cisplatin-resistant ovarian cancer cells. Silencing of lncRNA PART1 led to cisplatin sensitivity in cisplatin-resistant ovarian cancer cells, which is shown by suppression of proliferation, invasion and migration (Yang et al., 2021). YY1 transcription factor can induce the expression of lncRNA PART1. PART1 can target miR-512-3p and regulate the expression of CHRAC1 in ovarian cancer cells. Taken together, lncRNA PART1 targeted miR-512-3p/CHRAC1 axis to increase cisplatin resistance in ovarian cancer (Yang et al., 2021).

### LncRNA UCA1

LncRNA UCA1 reduced cisplatin response of OAW42 ovarian cancer cells via direct sponging to miR-27a-5p and decreasing the expression of UBE2N levels, resulting in the inhibition of BIM expression, a member of the Bcl-2 family to induce apoptotic death (Wambecke et al., 2021). UCA1 accelerated cisplatin resistance via miR-27a-5p/UBE2N/BIM axis in ovarian cancer cells (Wambecke et al., 2021).

### LncRNA WDFY3-AS2

Evidence revealed that lncRNA WDFY3-AS2 participates in regulation of cisplatin sensitivity in ovarian cancer cells (Wu et al., 2021). In cisplatin-resistant A2780 ovarian cancer cells, WDFY3-AS2 had an increased expression. Depletion of WDFY3-AS2 blocked migration and invasion of cisplatin-resistant A2780 cells, but induced apoptosis and proliferation inhibition (Wu et al., 2021). Moreover, WDFY3-AS2 stimulated tumor-spheres in cisplatin-resistant A2780 cells. WDFY3-AS2 interacted with miR-139-5p and elevated the expression of SDC4 (Wu et al., 2021). Silencing of WDFY3-AS2 caused tumor growth reduction in xenografts. Hence, WDFY3-AS2 could regulate cisplatin resistance via targeting miR-139-5p/SDC4 axis in ovarian cancer (Wu et al., 2021). Deletion of LINC00152 elevated cisplatin sensitivity via upregulation of Bax and cleaved caspase-3, and downregulation of Bcl-2 in ovarian cancer (Zou and Li, 2019). Moreover, deletion of LINC00152 reduced the expression of several drug resistant genes, such as MDR1, MRP1 and GSTπ in ovarian cancer (Zou and Li, 2019).

### Other lncRNAs promote cisplatin resistance

LncRNA NEAT1 depletion abrogated the cisplatin resistance via modulation of miR-770-5p and PARP1 in ovarian cancer (Zhu et al., 2020). LncRNA NEAT1 overexpression sponged miR-770-5p and inhibited the expression of PARP1, leading to cisplatin resistance in ovarian cancer (Zhu et al., 2020). Tan et al. found that lncRNA CHRF downregulation inhibited EMT and inactivated STAT3 pathway and abolish cisplatin resistance via regulation of miR-10b in ovarian cancer cells (Tan et al., 2020). LncRNA TRPM2-AS stimulated cisplatin resistance via binding to miR-138-5p and releasing SDC3 mRNA, leading to ovarian cancer malignant progression (Ding et al., 2021). Knockdown of lncRNA PVT1 repressed tumor progression via targeting JAK2/STAT3/PD-L1 in cisplatin-resistant ovarian cancer cells (Chen et al., 2021). Silencing of MALAT1 accelerated cisplatin sensitivity in ovarian cancer by suppression of the Notch1 pathway and ABCC1 expression (Bai et al., 2018). Knockdown of MALAT1 upregulated Bax protein and downregulated Bcl-2 protein in ovarian cancer cells (Bai et al., 2018). Upregulation of lncRNA PVT1 increased cisplatin resistance via targeting apoptotic pathway in ovarian cancer (Liu et al., 2015). LncRNA EBIC also facilitated cisplatin resistance via modulating Wnt/β-catenin in ovarian cancer cells (Xu et al., 2018). Linc00161 upregulation led to cisplatin resistance via regulating miR-128 and MAPK1 in ovarian cancer cells (Xu et al., 2019). LncRNA ANRIL depletion enhanced cisplatin resistance via sponging let-7 and increasing HMGA2 in ovarian cancer cells (Miao et al., 2019).

## LncRNAs inhibit cisplatin resistance

### LINC01508

LINC01508 expression was decreased in ovarian cancer cells with cisplatin resistance and ovarian cancer patients with platinum resistance (Xiao et al., 2021). In addition, tumor size and platinum resistance were linked to lower expression of LINC01508. Upregulation of LINC01508 increased cisplatin sensitivity of ovarian cancer cells via the suppression of the Hippo signaling pathway (Xiao et al., 2021). This study indicated that LINC01508 could be a biomarker for prediction of platinum resistance in ovarian cancer patients.

### LINC01125 and LINC00312

LINC01125 promoted cisplatin sensitivity through targeting miR-1972 in ovarian cancer (Guo and Pan, 2019). Cisplatin-resistant ovarian cancer specimens had a lower expression of LINC01125. Increased LINC01125 retarded proliferation of ovarian cancer cells and induced the cisplatin cytotoxicity (Guo and Pan, 2019). LINC01125 interacted with miR-1972 and induced cell apoptosis pathways in ovarian cancer. Hence, LINC01125 could work as an antitumor lncRNA to increase cisplatin sensitivity of ovarian cancer (Guo and Pan, 2019). Linc00312 overexpression increased the cisplatin sensitivity via regulation of the Bcl-2 and caspase-3 pathways in cisplatin-resistant ovarian cancer cells (Zhang et al., 2018).

### LncRNA GAS5

Using microarray and RT-PCR analysis, one group found that lncRNA GAS5 was dramatically downregulated in ovarian cancer specimens (Long et al., 2019). Low expression of lncRNA GAS5 was associated with poor prognosis in patients with ovarian cancer. In line with the role of GAS5, cisplatin-resistant ovarian cancer cells displayed lower expression of lncRNA GAS5 (Long et al., 2019). Increased expression of lncRNA GAS5 led to G0/G1 phase arrest and triggered apoptosis in ovarian cancer cells. Notably, overexpression of lncRNA GAS5 elevated the cisplatin sensitivity in ovarian cancer cells and in mice. LncRNA GAS5 recruited E2F4 to PARP1 and modulate the activation of MAPK pathway, leading to inhibition of cisplatin resistance (Long et al., 2019).

### LncRNA ENST00000457645

One study showed that lncRNA ENST00000457645 reduced cisplatin resistance in ovarian cancer cells (Yan et al., 2017). LncRNA ENST00000457645 overexpression suppressed viability and migratory capacity of ovarian cancer. This lncRNA upregulation increased the expression of Bax protein and cleaved caspase-3 in CP70 ovarian cancer cells (Yan et al., 2017). Further investigation is pivotal for determining the mechanism of lncRNA ENST00000457645-mediated cisplatin resistance.

## LncRNAs regulate carboplatin resistance

### LncRNA SNHG12

RNA sequencing and global DNA methylation assays were performed in four carboplatin-sensitive ovarian cancer cell lines and their resistant cells, and two ovarian cancer cells (OVCAR8 and Ovc316) with inherent carboplatin-resistant cell lines (Abildgaard et al., 2022). TCGA dataset and internal database were used to validate lncRNA candidates. This study found that 4,255 DEGs and 14,529 DMPs (differentially methylated CpG positions) in carboplatin-resistant cells (Abildgaard et al., 2022). They found that 50 lncRNAs were linked to carboplatin resistance. Moreover, 11 lncRNAs exhibited DMPs, including lncRNA SNHG12 (Abildgaard et al., 2022). Furthermore, depletion of lncRNA SNHG12 promoted carboplatin resistance in OVCAR8 and Ovc316 cells. Therefore, lncRNA SNHG12 accelerated carboplatin resistance in ovarian cancer (Abildgaard et al., 2022).

### LncRNA TLR8-AS1

TLR8-AS1 knockdown suppressed cell migration and invasion, and reduced resistance to carboplatin in OV90 and SKOV3 (Xu et al., 2020). TLR8-AS1 can stabilize TLR8 mRNA and increase the expression of TLR8, leading to activation of NF-κB signaling pathway (Xu et al., 2020). TLR8-AS1 expression level was upregulated in ovarian cancer tissues, especial in metastatic ovarian cancer, which was linked to poor prognosis. Altogether, TLR8-AS1 targeted NF-κB pathway to govern tumor metastasis and carboplatin resistance (Xu et al., 2020).

### LncRNA HOTAIR

LncRNA HOTAIR expression was linked to poor survival in ovarian cancer patients with carboplatin treatment (Teschendorff et al., 2015). HOTAIR and its DNA methylation file suggest carboplatin resistance in ovarian cancer patients (Teschendorff et al., 2015). However, it is necessary to determine and find direct evidence to demonstrate the function of HOTAIR in carboplatin resistance in ovarian cancer.

## LncRNAs regulate paclitaxel resistance

### LncRNA HOTAIR

LncRNA HOTAIR was identified to play an important role in ovarian carcinogenesis.

HOTAIR facilitated paclitaxel resistance by regulation of checkpoint kinase 1 (CHEK1) in ovarian cancer (Jiang et al., 2020b). Paclitaxel treatment increased the expression of HOTAIR in ovarian cancer cells. Moreover, downregulation of HOTAIR suppressed cell proliferation and enhanced paclitaxel sensitivity as well as caused G2/M phase arrest in ovarian cancer cells (Jiang et al., 2020b). Overexpression of CHEK1 weakened HOTAIR knockdown-induced paclitaxel sensitivity in ovarian cancer (Jiang et al., 2020b).

### LncRNA SNHG5

Lin et al. reported that lncRNA SNHG5 promoted paclitaxel sensitivity via repressing the expression of miR-23a in ovarian cancer cells (Lin et al., 2020). SNHG5 was remarked downregulated in patients with ovarian cancer and associated with poor prognosis. SNHG5 can act as a decoy to repress miR-23a in ovarian cancer cells (Lin et al., 2020). Knockdown of miR-23a or overexpression of SNHG5 overcame the paclitaxel resistance in ovarian cancer cells (Lin et al., 2020).

### LncRNA SNHG7

LncRNA SNHG7 has been revealed to govern ovarian tumorigenesis. For instance, lncRNA SNHG7 can be activated by SP1 and interact with EZH2 and confer oncogenic functions in ovarian cancer (Bai et al., 2020). LncRNA SNHG7 knockdown by siRNA transfection retarded migration and invasion of paclitaxel-resistant cells in ovarian cancer (Zhang et al., 2021). LncRNA SNHG7 interacted with EIF4G2 and affected the degradation of EIF4G2, leading to upregulation of EIF4G2. LncRNA SNHG7 promoted cell viability and motility as well as paclitaxel resistance via promotion of EIF4G2 (Zhang et al., 2021).

### LncRNA SDHAP1

LncRNA SDHAP1 had a higher expression in paclitaxel-resistant Hey-8 and SKOV3 cells (Zhao et al., 2020). Depletion of lncRNA SDHAP1 stimulated paclitaxel sensitivity in ovarian cancer cells. Mechanically, lncRNA SDHAP1 sponged miR-4465 and promoted the expression of EIF4G2, which conferred paclitaxel-induced cell apoptotic death in ovarian cancer cells (Zhao et al., 2020).

### LncRNA MIR17HG

LncRNA MIR17HG upregulation inhibited paclitaxel resistance and glycolysis via regulation of Claudin in epithelial ovarian cancer (EOC) cells (Wu et al., 2022b). Moreover, KHDRBS3 interacted with MIR17HG and blocked the function of MIR17HG in EOC cells (Wu et al., 2022b). KHDRBS3 expression was increased in paclitaxel-resistant EOC cells, while KHDRBS3 downregulation reduced the IC50 of paclitaxel-resistant paclitaxel in EOC cells. MIR17HG overexpression abrogated the KHDRBS3-mediated paclitaxel-resistance in EOC cells (Wu et al., 2022b).

### LncRNA MALAT1

Overexpression of lncRNA MALAT1 led to promotion of cyclin D1, pAkt and p-PI3K, contributing to acceleration of ovarian cancer cell proliferation (Mao et al., 2021). MALAT1 elevated the expression of IL-1β, p-P38, p-NF-κB, COX2 and PGE2 signaling, and reduced caspase-3 level and promoted Bcl-2 expression in ovarian cancer cells. MALAT1 upregulation also elevated the expression of ZEB2, YAP, vimentin and decreased E-cadherin, suggesting that MALAT1 triggered EMT and tumor metastasis in ovarian cancer cells (Mao et al., 2021). Moreover, MALAT1 upregulation increased paclitaxel resistance of ovarian cancer cells in the tumor microenvironment (Mao et al., 2021).

### LncRNA UCA1

LncRNA UCA1 has been reported to enhance paclitaxel resistance via targeting the miR-654-5p and SIK2 in ovarian cancer (Li et al., 2020a). LncRNA UCA1 upregulation was found in ovarian cancer tissues and paclitaxel-resistant ovarian cancer cells (Wang et al., 2018b; Li et al., 2020a). Paclitaxel resistance was restrained by inhibition of lncRNA UCA1 in paclitaxel-resistant ovarian cancer cells (Li et al., 2020a). Moreover, UCA1 sponged the miR-654-5p and upregulated the expression of its target SIK2 in ovarian cancer cells (Li et al., 2020a). In line with this finding, downregulation of SIK2 blocked paclitaxel resistance and attenuated the phenomenon of paclitaxel-resistant ovarian cancer cells, but inhibition of miR-654-5p by inhibitors rescued this inhibitory phenotype (Li et al., 2020a). Another study revealed that knockdown of UCA1 sensitized the paclitaxel-resistant ovarian cancer cells to paclitaxel treatment via induction of apoptosis (Wang et al., 2018b). Mechanistically, UCA1 can sponge miR-129 and increase the expression of ABCB1, contributing to paclitaxel resistance in ovarian cancer (Wang et al., 2018b).

### LncRNA KB-1471A8.2

LncRNA KB-1471A8.2 upregulation reduced proliferation and migratory ability of ovarian cancer cells (Zhang et al., 2019). Increased expression of lncRNA KB-1471A8.2 also antagonized the paclitaxel resistance in ovarian cancer cells (Zhang et al., 2019). KB-1471A8.2 was decreased in ovarian cancer specimens and chemo-resistant ovarian cancer cell lines. KB-1471A8.2 upregulation can inhibit the expression of CDK4 in ovarian cancer cells, leading to G0/G1 phase arrest (Zhang et al., 2019).

### LncRNA PRLB

LncRNA PRLB was found to regulate the paclitaxel resistance in ovarian cancer cells (Zhao and Hong, 2021). Paclitaxel-resistant ovarian cancer samples and cell lines exhibited the increased expression of lncRNA PRLB. Silencing of lncRNA PRLB reduced the IC50 value of the paclitaxel-resistant SKOV3 and CAOV3 ovarian cancer cells to paclitaxel treatment due to upregulation of apoptosis. LncRNA PRLB can bind to miR-150-5p and suppress the expression of miR-150-5p in ovarian cancer cells (Zhao and Hong, 2021). RSF1 was further identified as a target of miR-150-5p in ovarian cancer cells. RSF1 can activate the NF-κB signaling pathway in ovarian cancer cells (Zhao and Hong, 2021). Hence, lncRNA PRLB enhanced paclitaxel resistance via targeting miR-150-5p/RSF1/NF-κB in ovarian cancer cells.

### Other lncRNAs regulate paclitaxel resistance

LncRNA SNHG1 knockdown enhanced paclitaxel sensitivity in A2780 cells via suppression of cell growth and migration and induction of apoptosis by regulating miR-216b-5p (Pei et al., 2020). LINC01118 plays an oncogenic role in promotion of paclitaxel resistance via modulation of miR-134 and upregulation of ABCC1 in ovarian cancer cells (Shi and Wang, 2018).

## LncRNAs regulate 5-FU and rapamycin resistance

LncRNA TMPO-AS1 was reported to take part in 5-fluorouracil resistance in ovarian cancer cells (Li et al., 2020b). LncRNA TMPO-AS1 governed the expression of the TMEFF2 via sponging miR-200c, and activated the PI3K/Akt pathway. Depletion of TMPO-AS1 suppressed the EMT and motility and 5-FU resistance in ovarian cancer cells (Li et al., 2020b). LncRNA EPIC1 enhanced rapamycin resistance via activation of AKT-mTORC1 pathway in ovarian cancer (Wang et al., 2020b). Myc was involved in EPIC1-mediated oncogenesis in ovarian cancer cells. Overexpression of lncRNA EPIC1 activated the AKT-mTORC1 pathway via Myc and caused rapamycin resistance in ovarian cancer (Wang et al., 2020b).

## Conclusion and perspective

**FIGURE 1:**
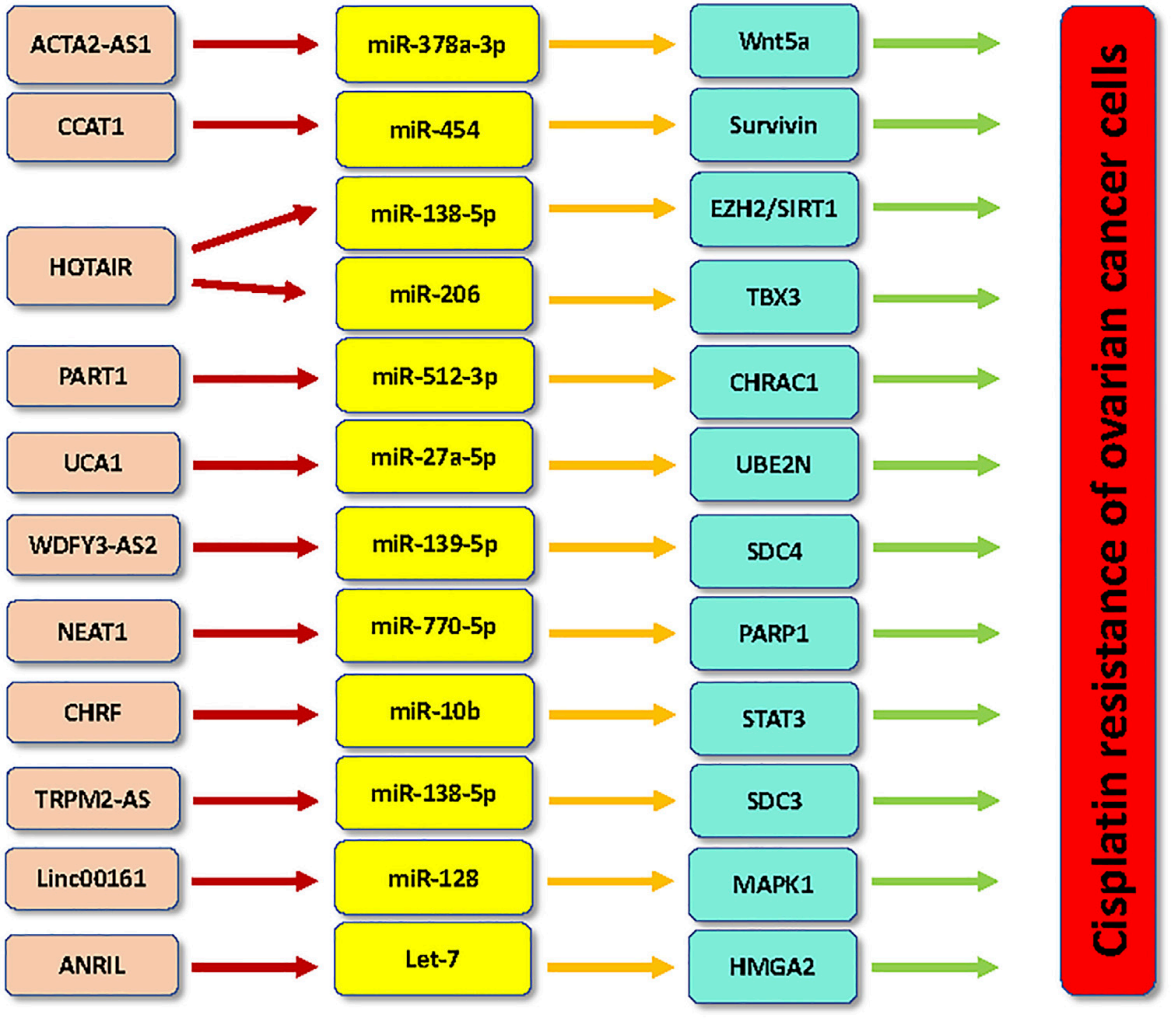
LncRNAs regulate the cisplatin resistance in ovarian cancer.

**FIGURE 2:**
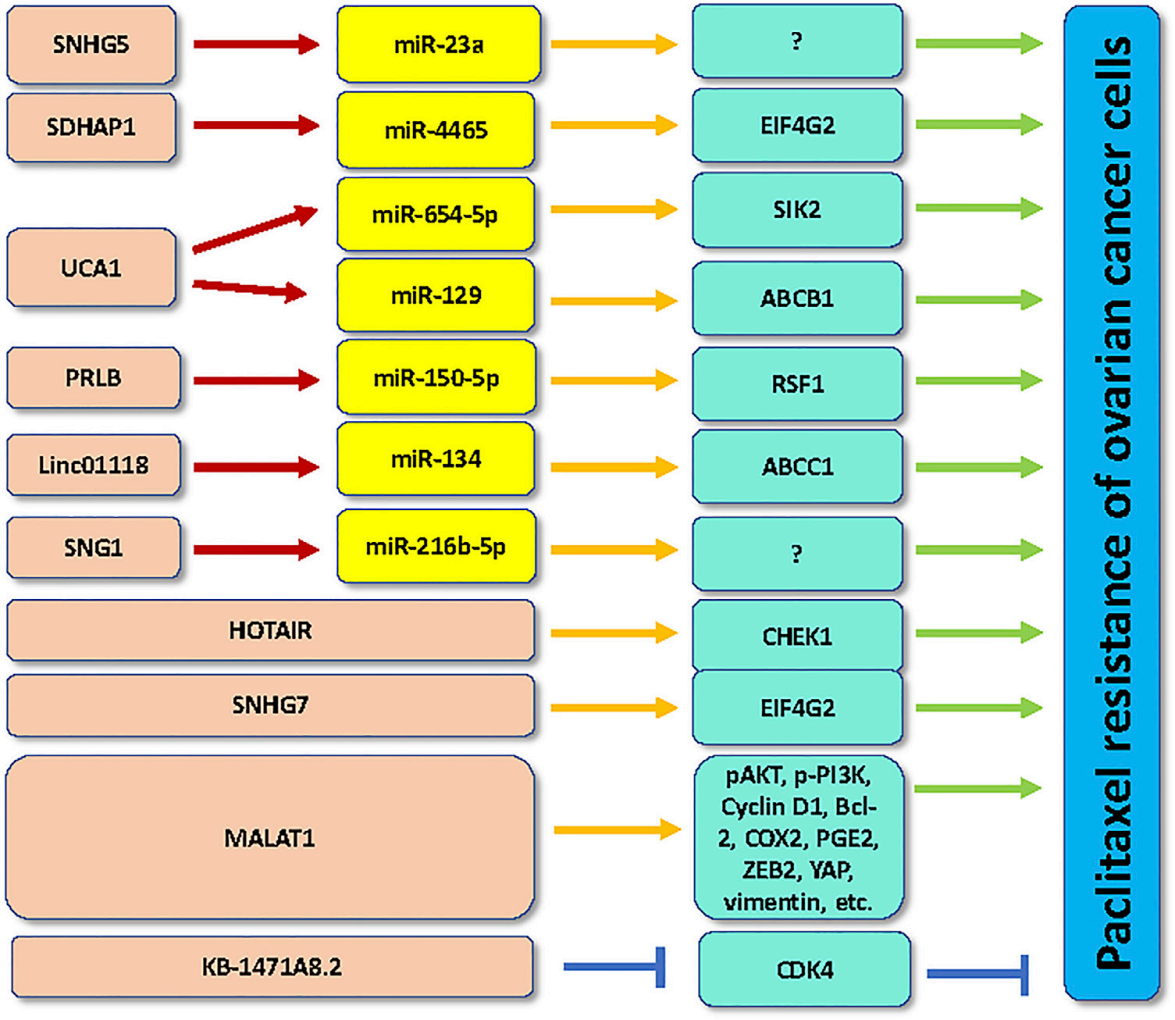
LncRNAs regulate the paclitaxel resistance in ovarian cancer.

In conclusion, lncRNAs regulate chemoresistance via sponging miRNAs in ovarian cancer cells (Figures 1, 2). It has several issues that need to be mentioned. Firstly, evidence suggests that cicrRNAs also regulate chemoresistance in ovarian cancer. For instance, silencing of circNRIP1 enhanced the sensitivity of paclitaxel in ovarian cancer cells through regulation of miR-211-5p and homeobox C8 (HOXC8) pathways (Li et al., 2020c). Paclitaxel-resistant ovarian cancer tissues and cells exhibited the higher expression of circNRIP1 (Li et al., 2020c). Knockdown of circNRIP1 abolished the paclitaxel resistance in ovarian cancer cells and mice. Furthermore, circNRIP1 interacted with miR-211-5p and subsequently increased the expression of HOXC8 in ovarian cancer cells (Li et al., 2020c). One study demonstrated that increased expression of circ_CELSR1 enhanced paclitaxel resistance via targeting miR-149-5p/SIK2 axis in ovarian cancer (Wei et al., 2021). Consistently, silencing of circ_CELSR1 reduced resistance of ovarian cancer cells to paclitaxel treatment (Wei et al., 2021).

Secondly, several compounds have been reported to target the expression of lncRNAs to control drug resistance in ovarian cancer. For example, Metformin inhibited the expression of lncRNA SNHG7 and increased the miR-3127-5p expression levels as well as regulated autophagy, which increased paclitaxel sensitivity (Yu et al., 2020). Curcumin elevated the lncRNA MEG3 levels via demethylation of MEG3, leading to inhibition of extracellular vesicle-involved transfer of miR-214, thereby attenuation of cisplatin resistance in ovarian cancer (Zhang et al., 2017). Thirdly, one lncRNA can regulate resistance of several chemotherapeutic drugs in ovarian cancer. For example, lncRNA ZEB1-AS1 attenuated cisplatin and paclitaxel sensitivity in epithelial ovarian cancer cells via suppression of MMP19 (Dai et al., 2021). LncRNA HOTAIR regulated cisplatin resistance and paclitaxel resistance in ovarian cancer cells (Zhang et al., 2020a; Jiang et al., 2020b). LncRNA MALAT1 facilitated both cisplatin and paclitaxel resistance in ovarian cancer cells via inhibition of cell apoptosis and promotion of invasion and proliferation (Mao et al., 2021). Taken together, multiple lncRNAs regulate the drug resistance in ovarian cancer. Due to that lncRNAs are involved in drug resistance in ovarian cancer, targeting these lncRNAs could be useful strategy for overcoming chemoresistance in ovarian cancer patients.

## Abbreviations

CCAT1, colon cancer-associated transcript 1; CircNRIP1, circRNA nuclear receptor-interacting protein 1; EIF4G2, eukaryotic translation initiation factor 4 gamma 2; HOTAIR, Hox transcript antisense intergenic RNA; KHDRBS3, KH domain-containing, RNA-binding signal transduction-associated protein 3; NEAT1, nuclear paraspeckle assembly transcript 1; PANDAR, the promoter of CDKN1A antisense DNA damage activated RNA; SDHAP1, succinate dehydrogenase complex flavoprotein subunit A pseudogene 1; SFRS2, arginine/serine-rich 2; SIK2, salt inducible kinase 2; TMEFF2, transmembrane protein with epidermal growth factor and two follistatin motifs 2; UCA1, urothelial carcinoma associated 1.
